# The evolution of an SBNS-accredited NANSIG simulated skills workshop for aspiring neurosurgical trainees: an analysis of qualitative and quantitative data

**DOI:** 10.1007/s00701-020-04325-6

**Published:** 2020-05-01

**Authors:** Melissa Gough, Georgios Solomou, Danyal Zaman Khan, Mohammed Kamel, Daniel Fountain, Ashwin Kumaria, Richard Ashpole, Saurabh Sinha, Nigel Mendoza

**Affiliations:** 1 School of Medical Education, Newcastle University Medical School, Framlington Place, Newcastle upon Tyne, NE2 4HH UK; 2 Keele University Medical School, Stoke-on-Trent, UK; 3 Academic Neurosurgery Department, University of Cambridge, Cambridge, UK; 4 National Hospital for Neurology and Neurosurgery – UCLH, London, UK; 5 Department of Neurosurgery, Salford Royal NHS Foundation Trust, Salford, UK; 6 Nottingham University Hospitals NHS Trust – Queen’s Medical Centre Campus, Nottingham, UK; 7 Sheffield Teaching Hospitals, Sheffield, UK; 8 Imperial College Healthcare NHS Trust, Charing Cross Hospital, London, UK

**Keywords:** Simulation, Neurosurgery, Training, Improvement

## Abstract

**Background:**

The Neurology and Neurosurgery Interest Group (NANSIG) neurosurgical skills workshop is novel in teaching neurosurgical skills solely to medical students and foundation trainees in the UK. The aim is to offer an affordable option for a high-fidelity simulation course enabling students to learn and practise specific neurosurgical skills in a safe, supervised environment.

**Methods:**

A 10-delegate cohort was quantitatively assessed at the NANSIG neurosurgical skills workshop. Two assessors used a novel modified Objective Structured Assessment of Technical Skills (mOSATS) assessment tool, comprising 5 domains ranked according to a 5-point scale to rate delegates’ ability to create a burr hole. Qualitative data from previous workshops were collected, consisting of open-ended, closed-ended and 5-point Likert scale responses to pre- and post-workshop questionnaires. Data were analysed using SPSS® software.

**Results:**

Delegates scored a mean total of 62.1% (21.75/35) and 85.1% (29.8/35) in pre- and post-workshop assessments respectively revealing a statistically significant improvement. Regarding percentage of improvement, no significant difference was shown amongst candidates when comparing the number of neurosurgical cases observed and/or assisted in the past. There was no significant difference in the overall rating between the last two workshops (4.89 and 4.8 out of 5, respectively). One hundred percent of the attendees reported feeling more confident in assisting in theatre after the last two workshops.

**Conclusion:**

We show that a simulation workshop cannot only objectively quantify the improvement of surgical skill acquisition but can also be beneficial regardless of the extent of prior experience.

## Background

The National Neurology and Neurosurgery Interest Group (NANSIG) is the national student and junior doctor arm under the Society of British Neurological Surgeons (SBNS), offering opportunities for medical students and doctors who have not yet entered neurosurgical training. Amongst a plethora of activities, NANSIG organises and leads the delivery of an annual national neurosurgical skills workshop. Based on national feedback collated amongst students in the UK, almost 50% of students suggested that a neurosurgical skills workshop tailored to the medical students’ level of knowledge and expertise was lacking in the UK [20]. In a recent pan-European survey, a 13.4% overall rate of satisfaction with simulator training was reported [32]. In 2017, an attempt to address the unmet need was made. NANSIG, in collaboration with the SBNS, launched the first ‘Introduction to Neurosurgery’ skills workshop on 30 January 2017. Based on a literature review, the ROWENA® (Realistic Operative Workstation for Educating Neurosurgical Apprentices) simulator was chosen for the workshop [3] for its anatomical accuracy and tissue-handling capabilities resembling real/cadaveric tissue [18]. ROWENA® has previously been used at the ST1 Neurosurgical Bootcamp for external ventricular drain (EVD) insertion assessment using the Objective Structured Assessment of Technical Skills (OSATS) tool [6]. This identified better external ventricular drainage (EVD) placement accuracy as well as overall economy of movement. OSATS has been previously validated as a reliable tool for the assessment of surgical skills [24, 33] (Appendix 1).

The workshop represents the first of its kind, teaching neurosurgical skills solely to medical students and foundation trainees in the UK, and is SBNS-accredited as of its launch [20]. It was primarily designed to offer an affordable option for a high-fidelity simulated training experience, enabling delegates to learn and practise specific neurosurgical skills in a safe, supervised environment. For foundation year trainees, the primary aim was to allow those who are pursuing a career in neurosurgery to refine their skills and increase their confidence prior to the upcoming neurosurgical rotations. For medical students, the primary aim was to enable them to experience the practical side of neurosurgery to help them develop these skills, as well as to encourage enthusiasm for the neurosurgical specialty. To consolidate learning, teaching of fundamental aspects of clinical neuroscience underpinning the neurosurgical procedures complemented later workshops (17 November 2017, 20 June 2018, 18 June 2019). Since 2017, the workshops have been qualitatively evaluated and improved based on feedback.

However, during the most recent workshop (June 2019), we sought to use an objective, structured, skill-based assessment, aiming to quantitatively establish the effectiveness of an SBNS-endorsed NANSIG neurosurgical simulation workshop involving the use of the ROWENA® simulation model. For this purpose, we decided to use a novel modified Objective Structured Assessment of Technical Skills (mOSATS) assessment tool (Appendix 2), a truncated version of the original OSATS tool [6, 21, 27]. MOSATS was selected due to the requirement for a bespoke tool capable of recording the selected skill assessed during the workshop, burr holes. Additionally, we qualitatively assess its current and future use in terms of training and skill acquisition by collating and analysing feedback from questionnaires received to date.

## Methodology

### Design of qualitative and quantitative data collection

A mixed methods approach was used to analyse qualitative and quantitative data. Qualitative analysis involved detailing delegates’ responses from pre- and post-workshop questionnaires collated to date (17 November 2017, 20 June 2018 and 18 June 2019 (Appendices 4, 5, and 6)). The pre-workshop questionnaire comprised a series of questions regarding baseline demographics, prior exposure to neurosurgery and neurosurgical skills workshops. In addition, using 5-point Likert scale responses, attendees were asked to self-evaluate based on the workshop’s modules (Appendix 4). Furthermore, using 5-point Likert scale responses and forced choice/closed-ended questions, attendees were asked to evaluate the overall quality of the course via post-workshop questionnaire (Appendix 5). Lastly, during the last workshop, we distributed a simulation-specific questionnaire to evaluate the overall perception regarding its role and value (Appendix 6).

The qualitative data were based on data collated from 28 attendees during the last three workshops. Regarding qualitative data, we used a cross-sectional examination of the 10-delegate cohort to be compared with other cross-sectional qualitative data captured across the previous three NANSIG workshops. Internal validity was maintained through comparison of workshops conveying a standardised structure with regard to modules taught and core simulation equipment used. Quantitative data were collected using mOSATS to rank individual performance. The mOSATS assessment tool comprised a set of 7 domains ranked on a 5-point scale. Five out of 7 of the domains from the original OSATS [24] were retained, i.e. (i) respect of tissue, (ii) time and motion, (iii) instrument handling, (iv) flow of operation and forward planning and (v) knowledge of the procedure. Two out of 7 domains were excluded since they were not integrated within the course syllabus, i.e. (i) knowledge of instruments and (ii) use of assistants. We added two additional elements to the mOSATS. Firstly was a score for the overall impression to the examiner and secondly a score for the visual quality of the overall product. The rationale for the former was to help stratify delegate performance by assimilating assessment of skills such as forward planning with that of other domains. The latter is justified by the improved patient outcomes resulting from lack of bony fragments associated with the burr hole, which can cause post-operative pain and infection. The quantitative analysis was conducted based on data from 10 delegates attending the workshop on 18 June 2019 only.

### The workshop

#### Delegate selection

Delegates were selected using a triple-blind marking system, whereby NANSIG President, Vice President and Workshop Lead each marked the 28 applications received using a 10-point Likert scale system. This was based upon applicants’ commitment to neurosurgery and future vision for the field. The scores were then collated and ranked according to their mean score from the three markers. The top ten applicants were selected and invited to attend the workshop.

#### Workshop setup

**Fig. 1:**
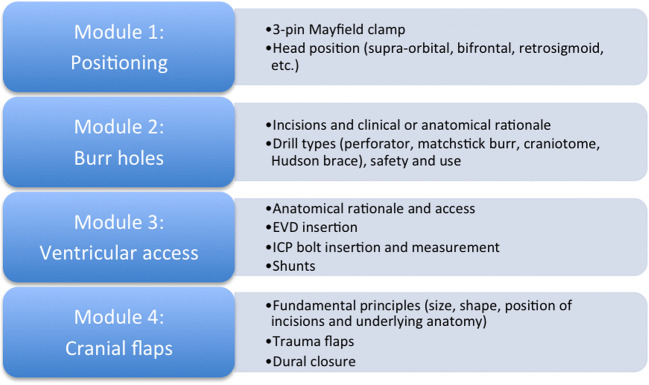
Workshop content

The Realistic Operative Workstation for Educating Neurosurgical Apprentices (ROWENA®) system was used at the workshop [3, 18]. Five workstations each included a ROWENA® simulator, 3-point head clamp and a drill. Other materials provided included cranial access kits and a range of neurosurgical instruments. Five neurosurgery registrars were invited to teach at the workshop, comprising trainees at a range of stages encompassing clinical medical student (CMS) to foundation year 2 trainees. There was a 2:1 student to teacher ratio. Teachers covered structured outcomes according to listed modules (Fig. 1).

### Quantitative assessment using mOSATS tool

Two assessment periods were undertaken, one pre- and one post-workshop teaching. A consultant neurosurgeon and an internal assessor, the registrar teachers, assessed each delegate. Delegates were assessed and scored during creation of a burr hole. Scoring domains included respect for tissue, time and motion, instrument handling, flow of operation and forward planning, knowledge of procedure, overall impression and quality of work (Appendix 2). The assessors used the mOSATS assessment scale provided independently. The use of two assessors’ averaged scores was intended to enhance validity.

### Qualitative assessment and feedback

A pre- and post-workshop questionnaire was distributed to delegates to collect responses concerning factors such as teacher and self-evaluation, course content, course administration and resource evaluation. Additional questions were added to workshop 2 and 3 questionnaires. See Appendices 4, 5 and 6 for pre- and post-workshop questionnaires and simulation-specific questionnaires, respectively.

### Safety considerations

A risk assessment was carried out in partnership with our main sponsor, Aesculap Academia and with Sheffield Teaching Hospitals NHS Foundation Trust. Contingency plans were made for any adverse events, which would be appropriately reported and followed up.

## Data management and statistical analysis

Data were kept on a secure, password-protected spreadsheet. Responses were anonymised. Data were extracted form Excel sheets (version) and statistical analysis was performed using IBM® SPSS® Statistics Version 24. A range of paired-sample *t* tests and one- and two-way ANOVA tests were applied to data to compare sets of workshop feedback and to reveal any statistically significant differences between workshops. Statistical significance was set at a *p* value of < 0.05.

## Results

### Modified OSATS assessment

**Fig. 2:**
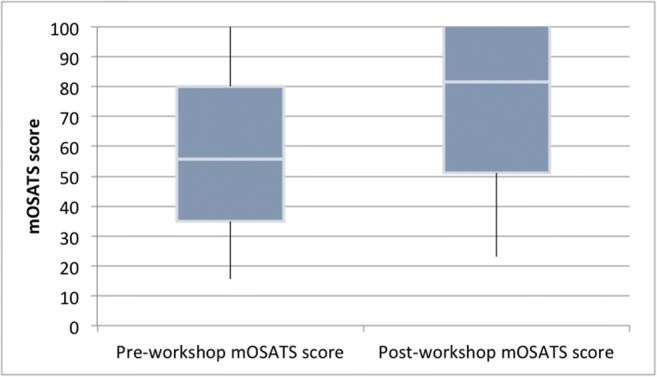
Pre- and post-workshop mOSATS score ranges

Ten delegates attended the workshop; four were foundation year doctors and six CMS. Delegates scored a mean total of 62.1% (21.75/35) and 85.1% (29.8/35) in pre- and post-workshop assessments, respectively (Table 1; Fig. 2). A 40.0% (8.05/35) average improvement was shown by the candidates between pre- and post-workshop assessment. A paired-sample *t* test was applied, and this revealed statistical significance between the two groups for this improvement (*t*(9) = − 7.667, *p* = 3.105). Two-way random intraclass correlation (ICC) analysis was performed on pre- and post-workshop assessment scores from the same pair of assessors per delegate, with a view to later generalisation using similarly skilled paired assessors in future studies. Pre- and post-workshop ICC analysis showed an average measure ICC of 0.534 with 95% confidence interval − 0.178–0.815, demonstrating moderate interrater reliability.

**Table 1:** Modified OSATS (mOSATS) results

Candidate	Training stage	Number of cases^*^	Pre-workshop mOSATS score				Post-workshop mOSATS score				Absolute improvement (points)
			Scorer 1	Scorer 2	Mean	%	Scorer 1	Scorer 2	Mean	%	
1	CMS^+^	21–50	24	14	19	54.3	29	29	29	82.9	10
2	CMS	11–20	20	14	17	48.6	27	29	28	80	11
3	FY2	1–10	21	21	21	60.0	31	29	30	85.7	9
4	FY1	11–20	21	35	28	80.0	31	35	33	94.3	5
5	FY2	11–20	22	35	28.5	81.4	28	34	31	88.6	2.5
6	CMS	1–10	27	18	22.5	64.3	29	25	27	77.1	4.5
7	CMS	1–10	22	9	15.5	44.3	29	17	23	65.7	7.5
8	FY1	11–20	29	21	25	71.4	31	33	32	91.4	7
9	CMS	11–20	20	20	20	57.1	28	35	31.5	90	11.5
10	CMS	1–10	22	20	21	0.6	32	35	33.5	95.7	12.5
Overall mean					21.75	62.1			29.8	85.1	8.05

*Assisting or observing

^+^Clinical medical student

**Fig. 3:**
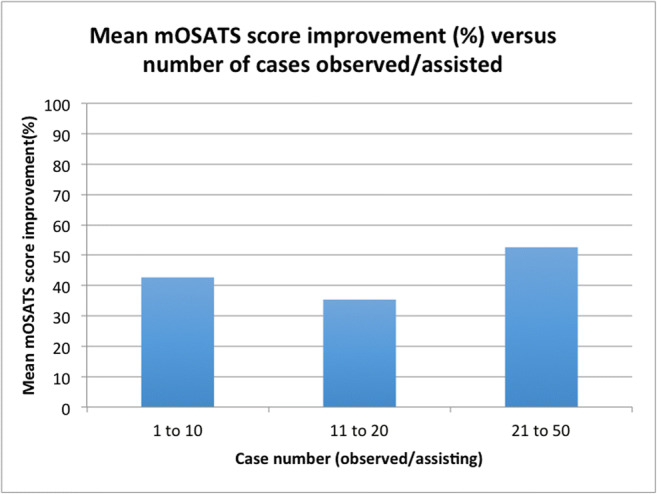
Mean mOSATS score improvement (%) versus number of cases observed/assisted

Delegates were divided into above or below average mOSATS score improvement groups, and further split into groups according to case number observed/assisted (Fig. 3). Paired-sample *t* test was used to compare candidates’ mOSATS scores according to improvement above (*t*(3) = 12.908, *p* = 0.001) or below (*t*(4) = 3.673, *p* = 0.021) mean improvement score with number of cases observed/assisted (Table 2). From the group who showed above average improvement (%), 2/5 assisted in 1–10 cases and 2/5 in 11–20 cases. From the group who showed below average improvement (%), 2/5 assisted in 1–10 cases and 3/5 in 11–20 cases. The below average 1–10 case group saw a 31.20% average increase, compared with a 18.21% average increase in the below average 11–20 case group. When attendees were split according to the number of neurosurgical cases observed/assisted, in the 1–10 case group, the average increase was 51.19%, compared with a 61.11% average increase in the above average improvement 11–20 case group. The single candidate with 21–50 case experience improved by 52.63%. A one-way ANOVA test revealed no statistically significant difference between case number and absolute mean improvement amongst the three groups (*p* = 0.830). See Fig. 3 for a breakdown of absolute improvement (%) stratified by number of cases observed/assisted.

### Qualitative self-evaluation prior to workshop

**Fig. 4:**
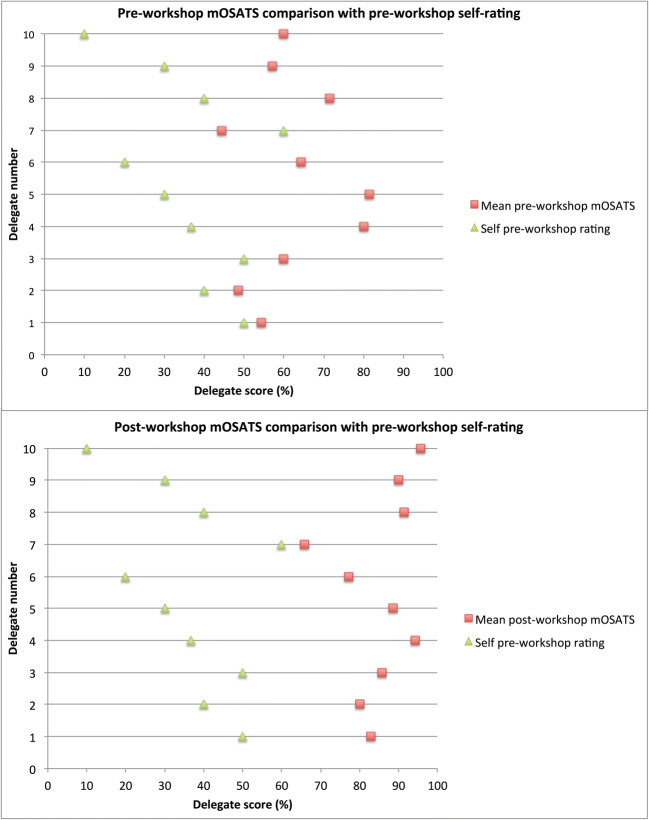
Comparison of pre-event questionnaire self-evaluation with mOSATS scores

Analysis of the pre-event questionnaire (see Table 2) shows a trend toward delegates ranking their knowledge as inversely proportional to their initial mOSATS score (Fig. 4).

## Qualitative feedback comparison

**Fig. 5:**
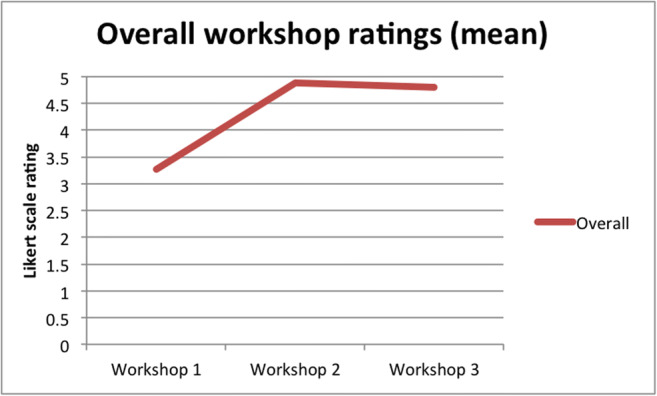
Overall mean workshop ratings using a 5-point Likert scale across three workshops (1: November 2017, Manchester; 2: June 2018, Sheffield; 3: June 2018, Sheffield)

Across the three workshops hosted by NANSIG, the overall ratings using 5-point Likert scale feedback (Appendices 4 and 5) from every delegate at each event show overall improvement and plateau at a high level (workshop 1 = 3.27 (*n* = 11); workshop 2 = 4.89 (*n* = 9); workshop 3 = 4.8 (*n* = 10); Fig. 5). A statistical analysis using a two-way ANOVA test showed that there is a statistically significant difference (*p* = 0.000) between the three sets of feedback compared. Further post hoc analysis, using a one-way ANOVA multiple comparisons test, revealed a statistically significant difference between workshop 1 versus workshops 2 and 3 (*p* = 0.001, *p* = 0.001). Furthermore, there is no statistically significant difference between overall mean workshop ratings between workshops 2 and 3 (*p* = 0.834).

**Fig. 6:**
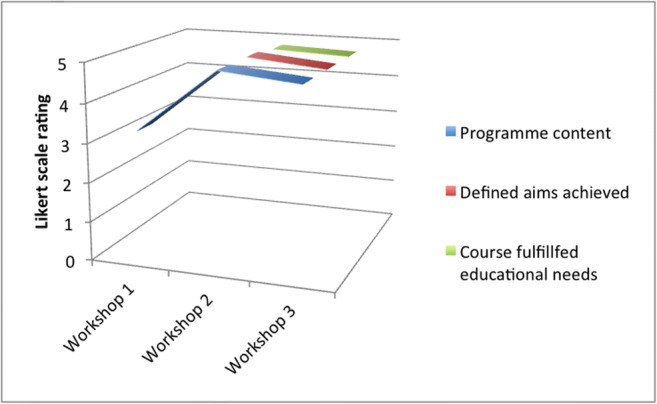
Delegate Likert scale (*y* axis: /5) responses from delegates at each workshop concerning programme content, whether educational aims were achieved, and whether the workshop fulfilled delegates’ educational needs

In response to programme content ranking using a 5-point Likert scale, ratings were 3.27, 4.89 and 4.8 for workshops 1, 2 and 3, respectively (Fig. 5). A two-way ANOVA test revealed a statistical significance (*p* = 0.001) in the comparison of these groups. Post hoc analysis, using a one-way ANOVA multiple comparisons test, demonstrated a statistically significant difference between both workshops 1 and 2 (3.27 and 4.89; *p* = 0.001), plus 1 and 3 (3.27 and 4.80; *p* = 0.003) (Fig. 6). There was no statistically significant difference between workshops 2 and 3 (4.89, 4.80, *p* = 0.506). There was no statistical significance in comparison using one-way ANOVA of workshops 2 and 3 regarding whether educational aims were achieved (4.89, 4.70, *p* = 0.341), educational aims were well-defined (4.89, 4.30; *p* = 0.062), delegates received educational fulfilment (4.78, 4.7, *p* = 0.434), programme content (4.89, 4.60, *p* = 0.171) nor delegates’ perceived value for money (4.67, 4.40; *p* = 0.506). In response to the question regarding whether delegates felt more confident in assisting in theatre after completing the workshop, 70% and 100% responded positively for workshops 1 versus 2 and 3, respectively. In response to the question regarding whether delegates would recommend the workshop to a friend, 70% and 100% responded positively for workshops 1 versus 2 and 3, respectively. Furthermore, whether delegates would be interested in a more advanced neurosurgical skills course, 80% and 100% responded positively for workshops 1 versus 2 and 3, respectively. The remaining standardised feedback questions (scored 1–5) concerned administration, infrastructure and resources and have been excluded from statistical analysis.

## Simulation-specific feedback

A post-workshop questionnaire was sent out to delegates to gather their opinions on the role of simulation in training and national selection process. Eight out of 10 (80%) responses were received and all delegates (100%) agreed that simulation has a role in neurosurgical training. Themes emerging from free-text responses included the value of practising skills in a safe, controlled environment before transferring these skills to patients, as well as the removal of time constraints that may be present in real-world scenarios.

“Yes, because the delegates can focus on improving skills in a stress-free environment and maybe they can focus on improving the mistakes without the pressure of time. At the same time, patient safety is ensured.”

“Absolutely. Given high stakes nature of even simple neurosurgical interventions the ability to familiarise yourself with equipment and basic procedural aspects in a safe environment is invaluable.”

**Fig. 7:**
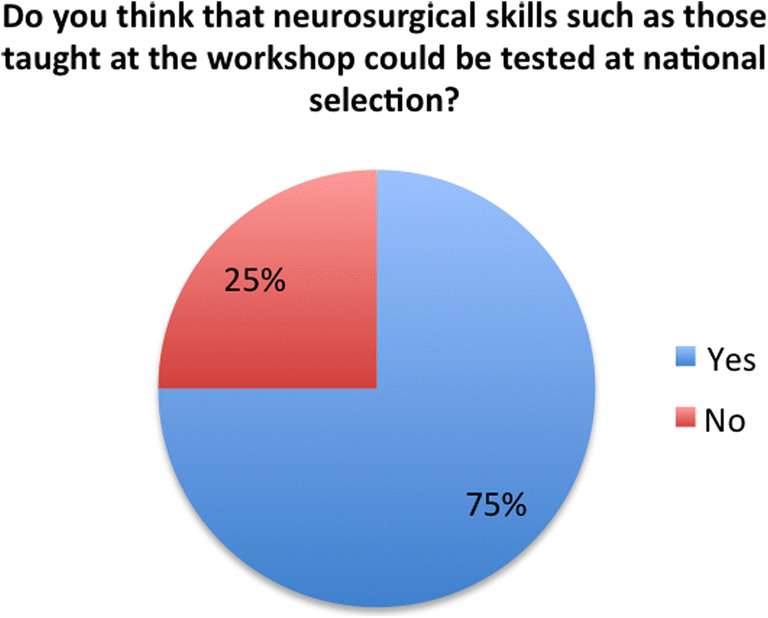
Delegates’ opinions on the use of workshop skills in testing at national selection

Delegates were asked for their opinions on the inclusion of courses such as this neurosurgical skills workshop in the desirable section of the ST1 neurosurgical personal specification criteria. 87.5% of the sample responded positively to this suggestion, although a sizeable minority (37.5%) of those delegates responding positively to the question indicated a predilection for a caveat that courses such as the NANSIG/SBNS workshop continue to be subsidised in order to make them affordable for student applicants. Finally, delegates were asked outright if they thought that the skills taught in the modules on this course could be tested at national selection of which 75% responded positively (Fig. 7).

## Discussion

The workshop has proven to be a valuable training experience in skill acquisition. Regarding quantitative analysis, attendees have shown a 23% overall improvement in mOSATS score before versus after training. To ascertain whether experience affects the overall improvement in performance (pre- versus post-workshop scores), an average mOSATS improvement (%) score across all attendees was calculated. Thereafter, delegates were initially categorised into ‘above average’ versus ‘below average’. We showed that prior experience in assisting/observing in theatre does not predict the extent of improvement in skill acquisition, indicated by the equally distributed number of cases assisted/observed in theatre between the two groups. To validate this even further, candidates were categorised into the number of neurosurgical cases observed and/or assisted in the past. No statistically significant difference in overall improvement was shown between the groups (1–10, 11–20 cases). This means that prior experience does not predict improvement in scores during workshop assessment, implying that such a workshop can be of benefit regardless of prior exposure in theatre. Indeed, the benefit of surgical simulation across experience grades is well-described in the literature [19].

To evaluate candidates’ ability to predict their own mOSATS scores, we asked them to rate their own skills prior to training. We found that their perceived scores compared with their actual scores prior to training were inversely proportional (Fig. 3). This shows that simulation training can potentially aid in highlighting weaknesses in delegates’ perceived abilities. The safe and controlled environment offers a platform to improve and reflect prior to performing these skills on real patients. There is, therefore, the potential to enhance patient safety as well as addressing the phenomenon of over-confidence in certain skill areas. Studies have demonstrated significant benefits in the use of surgical skills training in fostering more efficient and less error-prone performances amongst trainees [1]. Although lack of realism has been described as a limitation of simulation assessments [27], the aim of pre-junior workshop such as this is to practise skills in a safe and controlled environment.

In an attempt to reflect on the reliability of the mOSATS tool in the hands of the examiner, we assigned two examiners per candidate before and after training. The finding of moderate interrater reliability concerning the mOSATS tool will be investigated further through subsequent use of the scoring system during future workshops. The mOSATS scale was chosen because it can provide detailed information concerning five domains of assessment according to five scoring brackets. It is felt that this allows for sufficient differentiation of abilities within the cohort. Studies have proposed an objective system for measuring surgical skill although this employs that use of technology to track surgeons’ tool path, force applied and other metrics [14]. This was, however, used to assess microsurgical competencies, where more detailed measurements are required for accurate assessment. This novel mOSATS tool has proved for the first time that it can be used to quantitatively assess junior candidates in skill acquisition. We suggest that the mOSATS assessment tool was appropriate in this cohort since the skill assessed befits a junior trainee and the practical focus is suitable for a pre-junior trainee level.

Regarding long-term evolution, we have refined the modules and educational content over several workshops in order to obtain a now standardised core module offering. Refinement based upon troubleshooting issues from previous workshops, as well as delegate feedback, has been employed. This has involved adherence to four key fundamental skills (Fig. 1) and more coherence in their delivery. With the qualitative data collected using Likert scale rating, we have demonstrated that this is reflected in cohort feedback reflecting an averaged 31.6% increase in overall workshop ratings between the first and subsequent two workshops. There was no statistically significant difference in the qualitative comparison between the most recent two workshops. Unanimous responses stated that delegates would recommend the course to a friend, would consider an advanced course and felt more confident assisting in theatre as a result of these workshops. The response rates were less satisfactory, lying between 70 and 80%, in the case of the first workshop. This further suggests an upward trajectory in quality improvement across the entire workshop series. Standardising the workshop will now facilitate more statistically sound analysis in future studies concerning skills assessment and mOSATS validation.

It is one tenet of the workshop that there be an easily standardised way of teaching and assessing delegates in fundamental neurosurgical skills, thus enabling generalisation and easily reproducible methods. Further study involving a larger candidate cohort would be required to prove this hypothesis. The wider literature, however, does support our findings of improved post-simulation workshop scores. Numerous simulation courses have demonstrated improved post-test scores including those concerning endovascular [10, 22, 26] or spinal [12, 16, 31] neurosurgery, as well as management of CSF leaks during spinal surgery [13]. There was a 50.46% average increase in CMS group, compared with a 24.37% increase in FY trainees. This highlights the need for simulation in a safe environment particularly for more junior trainees. Challenging trainees’ responses to complications and unexpected anatomical variation during simulation workshops, therefore, should be championed. The competent acquisition of neurosurgical skills is essential when considering the potentially catastrophic events that may arise should a junior trainee make a mistake during a neurosurgical case. Similar improvement in objective assessments during simulation workshops was described in an analysis of data from 12 2-day courses held at Queen’s Medical Centre in Nottingham [4]. High-fidelity simulation training, incorporating core operative skills, has been provided in the form of the ST1 boot camp since its inception in 2014 [2]. There has been suggestion to develop further boot camp programmes at ST8 level, although no mention of provision of simulation training for earlier stage trainees and medical students. We propose that adding in simulation training at a more junior stage lays solid foundations in fundamental neurosurgical skills that can be further developed throughout training. This holds particular merit when considering the highly likely possibility of trainees at ST1 level undertaking basic operations. Simulation helps augment junior trainee experience by improving their surgical skills in the face of rising service demands.

The introduction of the European Working Time Directive to reduce working hours for doctors in training within the UK was aimed at improving patient safety and to improve working conditions [5]. A recent study found that this is at the detriment of continuity of patient care and reduced surgical training opportunities [25]. This translates to an increasing need to find alternative ways for neurosurgical skill acquisition for the benefit of junior surgeons in training. The use of simulation in training enables trainees to acquire essential neurosurgical skills in a safe environment, protected from the potentially devastating outcomes resulting from errors in high-precision procedures. A recent systematic review found that this use of simulation in training conveyed a range of qualitative and quantitative benefits such as improved tissue handling, accuracy and anatomical knowledge, amongst others [21]. A recent survey of SBNS members found that 65% of respondents agreed that simulation training should comprise part of the neurosurgical training curriculum [7]. The same survey found that simulation tools are not commonly available outside of teaching events with 85% of respondents having encountered simulation at such events, compared with 23% being exposed to simulation in hospitals [7]. This highlights the importance of workshops such as those offered by NANSIG and the need for expansion of their capacity and regularity. There is evidence that physical model simulation training resulted in improved technical skill in an operating theatre setting, enhancing the ability of trainees to develop their cognitive surgical expertise [29].

A notable physical model limitation is that of repetitive use, although a considerable advantage is the ability of trainees to handle physically a range of standard neurosurgical instruments and tools. This proprioceptive and true tactile learning input is not possible during the use of current VR systems. To address this, a recent European Association of Neurosurgical Societies course for early-stage trainees combined VR with cadaveric simulation [28]. This course employed a low tutor to delegate ratio and received excellent feedback on the small group learning with immediate feedback model similar to that of our workshop [28]. Conversely, another VR simulator study confirmed that simulator performance reflects surgeons’ ability to place an EVD correctly [30]. Examples of physical simulator models other than ROWENA include MARTYN [23], babyMARTYN [8], Sinus Model Oto-Rhino Neuro Trainer (SIMONT) [11] and the OMeR model (for simulation of deep microvascular anastomosis procedures) [17]. Qualitative and quantitative benefits from neurosurgical simulation have been widely described [9, 11, 15], although there exists a lack of standardised methodology and long-term follow-up within studies investigating the effects of simulation upon trainee skill acquisition and patient outcomes. Improved study design is recommended in order to generate higher quality generalisable data to contribute to a good evidence base concerning neurosurgical simulation.

## Conclusion

We show that using a mOSATS novel assessment tool, we can objectively quantify the improvement of surgical skill acquisition. Furthermore, a simulation workshop that has been refined based upon feedback can also be beneficial for skill acquisition regardless of the extent of prior experience.

## Limitations

The data series comprised a small cohort, largely due to the focus on low tutor to delegate ratios during teaching at the workshop. This format worked extremely well and NANSIG has continued to receive excellent feedback concerning course format hence the justification for this setup. Similarly, the number of assessors was limited within the study and reflects the scores of a consultant as well as trainees at various training stages. This could impact upon interrater reliability so future standardised briefing for assessors using mOSATS will be considered. Reliability of these results is optimised using robust methodology to generate a standardised and reproducible approach. The combination of qualitative and quantitative data adds contextual value to reinforce our conclusions. This will enable future studies to build upon these results in order to establish a rigorous and methodical way of assessing skill acquisition.

## Future work

Although the mOSATS tool will require validation for summative use, which can only be achieved through repetition and amassing a greater meta-cohort, the assessment is currently employed as a formative measure of performance. It is hoped that by standardising the modules taught and by delivering pre-assessment training regarding the mOSATS tool to assessors, this will also contribute to attainment of validity. Refinement of the mOSATS tool specific to neurosurgery may also benefit from being subjected to a Delphi process. Long-term follow-up will also be employed in order to achieve concurrent validity in comparing those scores attained during the simulation workshop with those in real operating theatre environments. Future studies will aim to compare performance in simulation workshop with that in real operative situations in order to obtain construct validity.

## Recommendations

Future simulation workshops must adapt a consistent application of standardised measurement instruments, as this is crucial for future pooled analysis. It is important that a standardised, validated assessment model such as a modified OSATS tool is established for comparison across different studies. Furthermore, gathering student/trainee opinions on the efficiency and appropriateness of neurosurgical simulation is vital so that suitable programmes can be developed and ultimately standardised to cater to their requirements. A consideration of the use of basic neurosurgical skills in a standardised format for use in national selection for speciality trainee neurosurgeons would be a natural progression once enough data has been gathered to validate the assessment of these skills. This could also have a positive impact upon speciality training itself.

## Appendix 2

### mOSATS scoring tool (/35)

#### Respect for tissue

1 - Frequently used unnecessary force on tissue or caused damage by inappropriate use of instruments

3 - Careful handling of tissue but occasionally caused inadvertent damage

5 - Consistently handled tissue appropriately with minimal damage

#### Time and motion

1 - Many unnecessary moves

3 - Efficient time/motion but some unnecessary moves

5 - Clear economy of movement and maximum efficiency

#### Instrument handling

1 - Frequently asked for the wrong instrument or used an inappropriate instrument

3 - Competent use of instruments although occasionally appeared stiff or awkward

5 - Fluid moves with instruments and no awkwardness

#### Flow of operation and forward planning

1 - Frequently stopped operating or needed to discuss next move

3 - Demonstrated some forward planning and reasonable progression of procedure

5 - Obviously planned course of operation with efficiency from one move to another

#### Knowledge of procedure

1 - Insufficient knowledge. Looked unsure and hesitant

3 - Knew all important steps of operation

5 - Demonstrated familiarity with all steps of the operation

Overall [/5]

Quality of final product [/5]

## Appendix 3

**Table 2:** Relationship of qualitative self-evaluation to absolute improvement in mOSATS scores

Candidate	Absolute improvement	Absolute improvement above mean	Absolute improvement below mean	Case mean (above mean absolute improvement)	Case mean (below mean absolute improvement)
1	10	10		35.5	
2	11	11		15.5	
3	9	9		4.5	
4	5		5		15.5
5	2.5		2.5		15.5
6	4.5		4.5		4.5
7	7.5		7.5		4.5
8	7		7		15.5
9	11.5	11.5		15.5	
10	12.5	12.5		4.5	
Overall mean	8.05	10.8	5.3	15.1	11.1

## Appendix 4. Pre-event questionnaire

What stage of training are you in?

- Pre-clinical medical
- Clinical medical student
- Intercalation
- Foundation year 1
- Foundation year 2

How many neurosurgical cases have you observed or assisted?

- 0
- 1–10
- 11–20
- 21–50
- 50+

Have you attended a course involving neurosurgical skills before?

- Yes
- No

All below criteria rated according to following scale:

1–5 (5 being excellent and 1 being very poor)

Module 1: Positioning

- Knowledge of 3-point head rest and pin use
- Knowledge of head positioning for neurosurgical procedures

Module 2: Burr holes

- Knowledge of burr hole incision
- Knowledge of burr hole positioning
- Knowledge of methods of burr hole drilling

Module 3: Ventricular access

- Knowledge of ICP measurement methods
- Knowledge of ventricular access positions
- Knowledge of technique of EVD insertion

Module 4: Flaps

- Knowledge of flap size and shapes
- Knowledge of closure techniques

## Appendix 5. Post-event questionnaire

How would you rate the overall course?

1–5 (5 being excellent and 1 being very poor)

Do you feel more confident about attending and assisting in theatre?

Yes/no

Would you recommend the course to a colleague?

Yes/no

Would you be interested in a more advanced neurosurgical course?

Yes/no

If yes to above, is there anything in particular you would like future courses to focus on?

Please rate the following areas of the course: (1 being poor and 5 being excellent)

- Registration process
- Venue
- Catering
- Demonstrators
- Value for money
- Programme content

Only in workshops 2 and 3:

- Aims of course were well-defined
- Defined aims achieved
- The course fulfilled my educational needs
- The course was well-organised
- What did you like about the course?
- What would you change and why?

## Appendix 6. Simulation-specific questionnaire

Only used for workshop 3:

- How useful did you fiend the pre-reading material?
  - 1–5 (5 being extremely useful and 1 being not at all useful)
- Have you ever received any virtual reality (VR) or physical model surgical simulation training before?
- Do you think simulation has a role in neurosurgical training?
- What do you think are the main barriers to simulation training?
- Do you think that neurosurgical skills such as those taught at the workshop could be tested at national selection?
- Do you think courses such as the one you attended should be added to the desirable criteria for the person specification for the ST1 (neurosurgery) national selection process?
